# A case report of autoimmune necrotizing myositis presenting as dysphagia and neck swelling

**DOI:** 10.1186/s12901-016-0027-3

**Published:** 2016-05-17

**Authors:** Linh Q. Ngo, Andrew G. Wu, Matthew A. Nguyen, Lauren E. McPherson, Elie Gertner

**Affiliations:** Division of Rheumatology, University of Minnesota Medical School, MMC 108 Mayo, 8108A, 420 Delaware St SE, Minneapolis, MN 55455 USA; Department of Internal Medicine, University of Minnesota Medical School, Minneapolis, MN USA; Section of Rheumatology, Regions Hospital, Saint Paul, MN USA

**Keywords:** HMGCR, Necrotizing myositis, Anti-3-hydroxy-3-methylglutaryl-coenzyme A reductase, Inflammatory myositis, Dysphagia

## Abstract

**Background:**

Severe dysphagia may occur in the immune mediated necrotizing myopathies (IMNM). Neck swelling and severe dysphagia as the initial symptoms upon presentation has not been previously described.

**Case presentation:**

A 55-year-old male with a 4 week history of neck swelling, fatigue, dysphagia, myalgias, night sweats, and cough was admitted for an elevated CK. He underwent extensive infectious and inflammatory evaluation including neck imaging and muscle biopsy.

Neck CT and MRI showed inflammation throughout his strap muscles, retropharyngeal soft tissues and deltoids. Infectious work up was negative. Deltoid muscle biopsy demonstrated evidence of IMNM. Lab tests revealed anti-3-hydroxy-3-methylglutaryl-coenzyme A reductase (HMGCR) antibodies confirming the diagnosis of HMGCR IMNM.

**Conclusions:**

HMGCR IMNM is a rare and incompletely understood disease process. Awareness of HMGCR IMNM could potentially lead to earlier diagnosis, treatment and improved clinical outcomes as disease progression can be rapid and severe.

## Background

The idiopathic inflammatory myopathies consist of rare autoimmune disorders that affect muscle as well as lung, skin, joints and other organ systems. They are characterized by marked symmetrical proximal muscle weakness. There is frequent CK elevation and electromyogram demonstrates a myopathy. While they often share clinical features, they can also have unique muscle biopsy findings. More recently it has been shown that approximately 60 % of patients with autoimmune myopathy have a myositis-specific autoantibody, each of which is associated with a distinct clinical phenotype. Thus future classification of these myopathies will include antibody patterns [1].

Immune mediated necrotizing myopathy (IMNM) has been recognized as a specific autoimmune myopathy that accounts for 19 % of all inflammatory myopathies [2]. In contrast to the idiopathic inflammatory myopathies which are characterized histopathologically by an inflammatory exudate, IMNM muscle biopsies show prominent fiber necrosis with minimal or no inflammation. Much attention has recently focused on IMNM associated with autoantibodies that recognize 3-hydroxy-3-methylglutaryl-coenzyme A reductase (HMGCR), the pharmacological target of statins [3]. HMGCR IMNM presents with symmetric proximal arm and leg weakness, myalgias, dysphagia and arthralgias [4]. While dysphagia occurs in about 20–35 % of patients [3, 5], prominent head and neck symptoms as the presenting complaint have not been described.

HMGCR IMNM has an aggressive and debilitating clinical course characterized by significant muscle necrosis. It is highly associated with current statin use, prior statin exposure and also can be idiopathic in a small number of cases but differs from statin induced myopathy [6–8]. Statin induced myopathy, is commonly characterized by myalgias with statin use that stop with discontinuation of the offending agent, and is a separate non-autoimmune entity [9]. On the other hand, HMGCR IMNM is an autoimmune disease that requires specific therapy and possibly long-term immunosuppression. HMGCR IMNM is associated with dysphagia in up to 35 % of patients, symmetric proximal muscle weakness in 95 %, distal muscle weakness in 41 %, and dyspnea in 37 %. CKs over 6000 and significant necrosis on muscle biopsy are commonly seen [6–8].

In 2011, a novel antibody to HMGCR was discovered in 6 % of a cohort of autoimmune inflammatory myositis patients (*n* = 750) at Johns Hopkins University School of Medicine [10]. Two-thirds of the patients had prior statin exposure. All patients with anti-HMGCR antibody IMNM demonstrated prominent necrosis with varying levels of inflammation on pathology. Statin-naïve patients were younger but the two subgroups were indistinguishable on pathology. Subsequent research supports the theory that this process is both due to genetic and environmental factors. Patients with HLA class II allele DRB1*11:01 have been shown to have an increased risk of developing HMGCR IMNM [11]. One possible model has been proposed by Mammen et al. HMGCR is an enzyme target of statins and has been shown to be upregulated in skeletal muscle cells that are exposed to statins in vivo [12]. Increased expression of the HMGCR triggers an immune response with formation of HMGCR antibodies and resultant skeletal muscle destruction and regeneration. In addition, regenerating muscle cells have also been demonstrated to increase expression of HMGCR [10] thereby potentiating the process over an extended period of time even after statin exposure is removed.

Treatment of anti-HMGCR IMNM requires several agents. Prednisone monotherapy is inadequate in up to 90 % of patients and most patients required 2 or more immunosuppressives [3]. Relapse rate is approximately 50–55 % in patients that taper off therapy [3, 6]. IVIG within the first three months of presentation has potentially promising outcomes with patients achieving significant improvement at 6 months [3, 13].

## Case presentation

A 55 year old African American male was admitted to the hospital with a 4 week history of difficulty swallowing, hoarseness, fatigue, neck swelling, night sweats and a 20 lb weight loss. He had no muscle weakness. He had type II diabetes mellitus and hyperlipidemia. His medications included atorvastain 40 mg daily which he was taking for 2 years. There was no history of rhabdomyolysis, or autoimmune disease.

On exam, he was afebrile with normal oxygen saturation on room air. His voice was notably hoarse and he had difficulty clearing his secretions. His neck was swollen symmetrically, non-tender, and without lymphadenopathy. Cardiovascular, pulmonary and gastrointestinal examination was unremarkable. Strength was 5/5 throughout, and he was able to stand and walk independently. There were no vasculitic skin lesions.

Basic hematological and chemistry studies were obtained and were within normal limits. His creatine kinase (CK) was 45,233 U/L (normal <120 U/L), AST was 835 U/L (normal <66 U/L), ALT was 277 U/L (normal <69 U/L) and aldolase was 83 U/L (normal <8.1 U/L). Basic autoimmune studies including anti-dsDNA, anti-Smith, anti-La, anti-Scl-70, anti-centromere, anti-cardiolipin, and anti-neutrophil cytoplasmic (ANCA) antibodies were all negative. Anti-Ro antibodies were positive. In addition, complement levels and immunoglobulin levels were normal. Infectious disease evaluations for all viral, fungal and bacterial etiologies were negative. Additional pertinent laboratory findings are displayed in Table 1. Video swallow study revealed significant oropharyngeal dysphagia.

**Table 1:** Pertinent lab values across management course

Day # of Management	1	5	8	20	48
Creatine Kinase (CK) (0–170 U/L)	28205	31986	45223	11246	2682
Anti-HMGCR Antibody (<20 U)	-	>200	-	-	104
Creatinine (0.66–1.25 mg/dL)	1.285	0.82	0.81	0.93	0.56
Erythrocyte Sedimentation Rate (ESR) (0–15 mm/h)	>68	49	-	-	-
C-Reactive Protein (CRP) (0.0–0.9 mg/dL)	7.4	3.8	3.3	-	-
Hemoglobin (13.5–17.5 g/dL)	14.6	-	-	14	11.0
Platelets (150–450 k/uL)	419	-	-	216	296
White Blood Count (4.0–11.0 k/uL)	9.1	-	-	32.4	10.4
Aspartate transaminase (AST) (0–66 U/L)	786	847	-	441	278
Alanine transaminase (ALT) (0–69 U/L)	268	304	-	336	374
Alkaline Phosphatase (38–126 U/L)	77	47	-	28	59
Bilirubin, Direct (0.0–.3 mg/dL)	0	0	-	0	0
Bilirubin, Total (0.2–1.3 mg/dL)	1.2	0.9	-	1.1	0.3

**Fig. 1:**
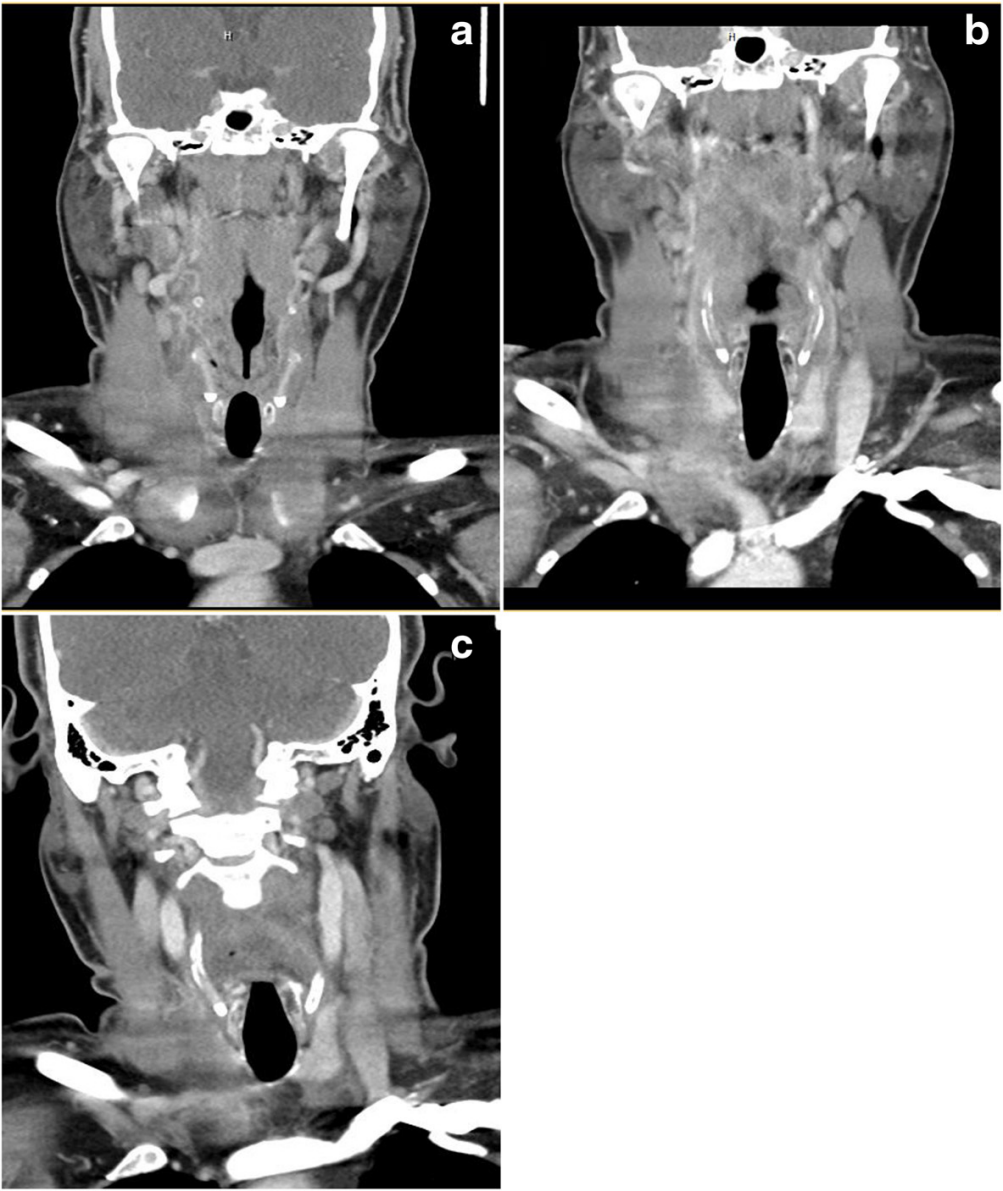
Neck CT Images. **a** Edema, stranding and exudates on initial presentation. **b** Worsening clinically after 4 days of antibiotic therapy. **c** Complete resolution 2 weeks after immunosuppressive therapy

Neck CT and MRI were suggestive of presumed infectious retropharyngeal exudate and phlegmon extending from the skull base to the upper thoracic region. There was diffuse inflammation of neck strap muscles (Fig. 1 panel a). Otolaryngology and infectious diseases were both consulted to assess for retropharyngeal abscess in the setting of severe dysphagia and neck soft tissue swelling. Broad spectrum antibiotics were started, however, the patient’s clinical status worsened. On the fifth day of admission, the patient developed weakness of the shoulders and an MRI of the shoulders revealed marked swelling, edema and inflammation of the deltoids and neck muscles. A muscle biopsy was performed of the left deltoid muscle and pathology revealed an inflammatory myopathy notable for myriad necrotic fibers, sometimes involving entire fascicles, many replaced by macrophages. An inflammatory exudate was present in perivascular regions in perimysium especially where the necrotic fibers were present. Myositis specific antibodies including Jo-1, Mi-2, PL-7, PL-12, SRP, EJ and OJ were negative. Subsequently, anti-HMGCR antibody testing was extremely positive at >200 U (normal <20 U).

He rapidly developed respiratory weakness with negative inspiratory force and vital capacity of -55 cm H2O (expected >80 cm H20) and 3.72 L (expected >3.8 L) respectively. This was followed by rapid and severe weakness of proximal upper and lower extremity muscles with inability to abduct his arms and stand up from seated position without assistance. He required a feeding tube for nutrition.

Treatment was initiated with IV methylprednisolone 500 mg daily for 3 days, which was followed by 5 days of plasmapheresis, followed by intravenous immunoglobulin (IVIG) therapy with 1 g/kg daily for 2 days.

**Fig. 2:**
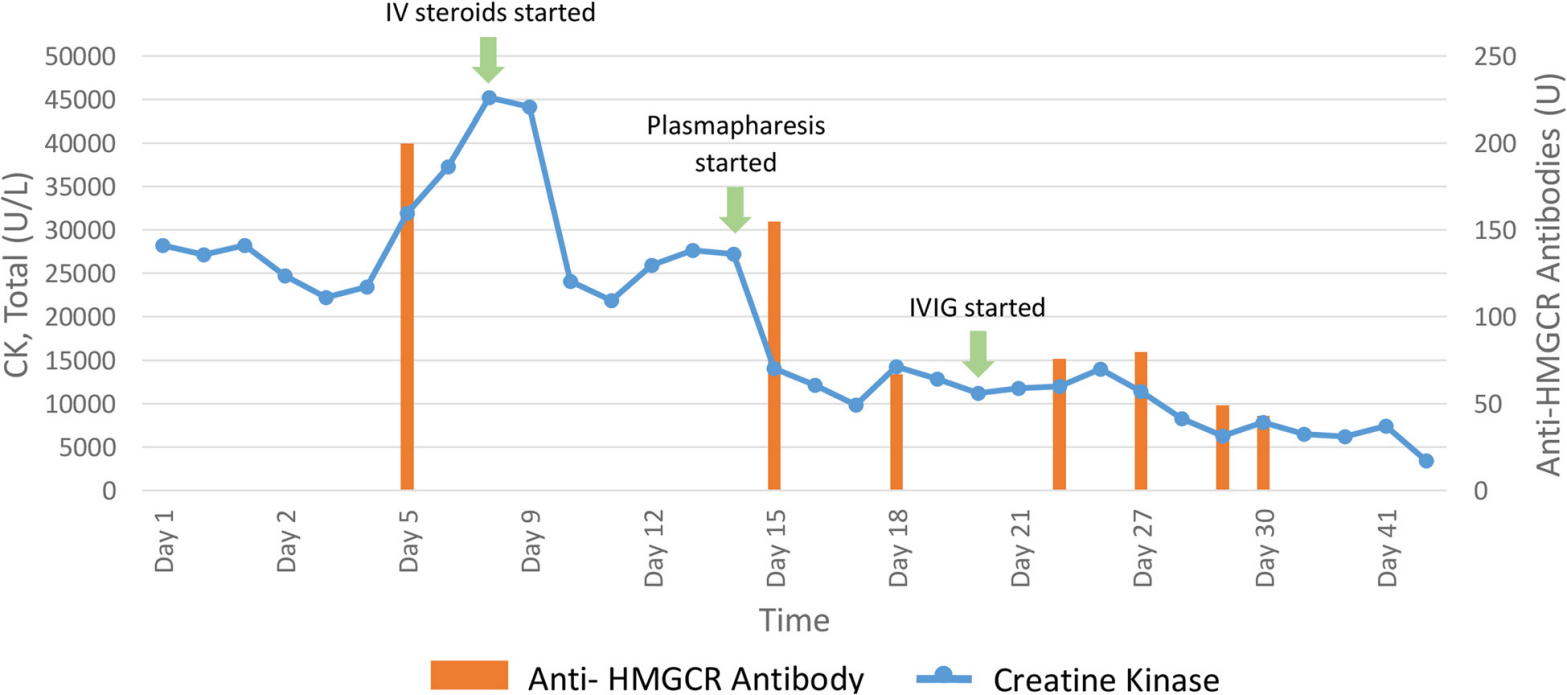
Creatine Kinase Levels and Anti-HMGCR Antibody Levels During Management

Follow-up CT scan of the neck after treatment revealed significant improvement in inflammation of strap muscles and retropharyngeal swelling (Fig. 1 panel b). The patient improved and was discharged home on only prednisone with plans for outpatient rehabilitation. Within 4 days of discharge, he was readmitted for worsening lower extremity weakness, darkening urine, and ongoing difficulties with managing secretions. He received additional IVIG 500 mg/kg daily for 2 days followed by initiation of rituximab 375 mg/m^2^ per week for 4 weeks and cyclophosphamide 15 mg/kg on weeks 1 and 3 as induction therapy. An MRI of the thighs confirmed ongoing muscle edema. His CK and anti-HMGCR antibody levels began to stabilize, and then improve as did his clinic symptoms. Figure 2 illustrates his CK and anti-HMGCR antibody levels in relation to his treatments. A repeat left thigh muscle biopsy showed a myriad of necrotic fibers, accompanied by a large number of regenerating fibers, all of which appeared to be in the same stage of regeneration confirming prior diagnosis of necrotizing myositis.

Subsequently, the patient was transferred to inpatient rehabilitation where he had progressive improvement in general fatigue, strength, and swallowing. He was discharged home requiring minimal assistance and after 3 months resumed all his usual activities. He was subsequently placed on methotrexate 15 mg weekly and a prednisone was tapered over the next 4 months without further complications.

## Conclusion

Prior to this report, no severe head and neck involvement has been described as the initial presentation of IMNM that closely resembled severe infection. Otolaryngologists may be consulted for head/neck involvement and severe dysphagia and should be aware of HMGCR antibody associated IMNM. Early recognition will allow for prompt initiation of immunosuppressive therapy and likely lead to better clinical outcomes.

## Declarations

### Ethics approval

NA.

### Consent for publication

Written informed consent was obtained from the patient for publication of this case report and any accompanying images. A copy of the written consent is available for review by the Editor of this journal.

### Availability of data and materials

NA.

## Abbreviations

HMGCR: 3-hydroxy-3-methylglutaryl-coenzyme A reductase
IMNM: immune mediated necrotizing myopathies
